# Guideline for antimicrobial treatment of multidrug-resistant Gram-negative infections: practice recommendations of the Brazilian Society of Infectious Diseases

**DOI:** 10.1016/j.bjid.2025.104589

**Published:** 2025-10-22

**Authors:** Alexandre Prehn Zavascki, Alberto Chebabo, Clovis Arns Cunha, Alexandre Rodrigues Silva, Gabriel Trova Cuba, Daniel Wagner C.L. Santos, Ana Cristina Gales

**Affiliations:** a Departamento de Medicina Interna, Universidade Federal do Rio Grande do Sul, Porto Alegre, RS, Brazil; b Serviço de Doenças Infecciosas, Hospital de Clínicas de Porto Alegre (HCPA), Porto Alegre, RS, Brazil; c Hospital Moinhos de Vento, Serviço de Doenças Infecciosas e Controle de Infecções, Porto Alegre, RS, Brazil; d Universidade Federal do Rio de Janeiro, Hospital Universitário Clementino Fraga Filho, Rio de Janeiro, RJ, Brazil; e Universidade Federal do Paraná, Curitiba, PR, Brazil; f Hospital Estadual Doutor Dório Silva, Serra, ES, Brazil; g Divisão de Doenças Infecciosas, Universidade Federal de São Paulo, São Paulo, SP, Brazil; h Departamento de Doenças Infecciosas, Universidade Federal do Maranhão, Hospital Universitário Presidente Dutra, São Luís, MA, Brazil; i Instituto D'Or de Pesquisa e Ensino, Hospital UDI, Rede D'Or, São Luís, MA, Brazil; j Antimicrobial Resistance Institute of São Paulo (ARIES), São Paulo, SP, Brazil

**Keywords:** Therapy, Carbapenems, *Pseudomonas aeruginosa*, *Acinetobacter baumannii*, *Enterobacterales*, Antimicrobial resistance

## Abstract

Although international guidelines are available, a national consensus is crucial to address the unique challenges faced in Brazil regarding the management of infections caused by multidrug-resistant Gram-negative bacilli (MDR-GNB). These challenges include marked regional disparities in antimicrobial access, variability in pathogen prevalence and resistance patterns, and unequal availability of diagnostic resources. This guideline, developed by a consensus of infectious diseases experts nominated by the Brazilian Society of Infectious Diseases, aims to support clinicians, particularly non-specialists, in the management of MDR-GNB infections across diverse healthcare settings in the country. The document focuses on pathogens classified as critical or high-priority by the World Health Organization (WHO), including Carbapenem-Resistant *Enterobacterales* (CRE), *Acinetobacter baumannii* (CRAB), and *Pseudomonas aeruginosa* (CRPA), ESBL- and AmpC-producing *Enterobacterales*, as well as *Stenotrophomonas maltophilia*, and *Burkholderia cepacia*. Therapeutic recommendations are organized by pathogen and infection site, including respiratory tract, skin and soft tissue, bloodstream, intra-abdominal, and both complicated and uncomplicated urinary tract infections.

## Introduction

Multidrug-resistant Gram-negative bacilli (MDR-GNB) pose a significant public health worldwide threat, particularly in Brazil, where alarming rates of resistance in major healthcare-associated pathogens have been documented.^1^^,^^2^ According to the latest report from the Brazilian Health Regulatory Agency (ANVISA), *Klebsiella pneumoniae* (4063 isolates; 17.0 %) and *Acinetobacter* spp. (2428 isolates; 10.1 %) were the second and fourth most frequently isolated pathogens causing central line-associated bloodstream infections in adult patients in Brazilian intensive care units (ICUs) in 2023.^1^ *Pseudomonas aeruginosa* (1703 isolates; 7.1 %) ranked just after *Enterococcus* spp. (1986 isolates; 8.3 %) and *Candida* spp. (1759 isolates; 7.3 %). Of particular concern are the high carbapenem resistance rates observed among these pathogens, with *Acinetobacter* spp. exhibiting the highest resistance rate (78.8 %), followed by *K. pneumoniae* (58.3 %) and *P. aeruginosa* (40.5 %).^1^ In a prospective, multicenter, observational study conducted in 14 hospitals across four of the five Brazilian regions between August 2022 and August 2023, carbapenem-resistant *K. pneumoniae* (CRKP) was the most common carbapenem-resistant *Enterobacterales* (CRE), representing 14.2 % of 1350 bloodstream infections (BSIs) isolates followed by carbapenem-resistant *A. baumannii* (CRAB), comprising 9.8 % of all isolates. Together, these two pathogens accounted for nearly a quarter of all BSI isolates, and nearly a third among BSIs of patients at ICUs.^2^ This critical scenario has prompted the Ministry of Health and ANVISA to emphasize the urgent need for effective interventions to mitigate the impact of MDR-GNB in Brazilian healthcare settings.^3^

Despite the existence of international guidelines,^4^, ^5^, ^6^, ^7^, ^8^, ^9^, ^10^, ^11^ a comprehensive national guideline is required to address specific challenges observed in Brazil, particularly the healthcare inequalities resulting in heterogeneous access to both older and newer antimicrobials. Regional variations in pathogen prevalence, antimicrobial resistance patterns, and disparities in diagnostic and therapeutic resources highlight the need for locally tailored recommendations. The objective of a national guideline is to improve the management of these highly prevalent difficult-to-treat healthcare associated infections in Brazil.

## Methods

### Panel selection

The Brazilian Society of Infectious Diseases board of directors selected Infectious Disease physicians with clinical and research experience in the treatment of MDR-GNB infections as panelists. Specialists from different Brazilian states and geographic regions were chosen to ensure that the diverse epidemiological patterns and local realities were considered in the development of this document.

### Process for recommendations

The choice of GNB-antimicrobial resistance selected for analysis of the panel has taken into account the 2024 World Health Organization (WHO) list of critical and high-priority GNB,^12^ with the addition of other difficult-to-treat GNB such as *S. maltophilia* and *B. cepacia.* The WHO list generally refers to third-generation cephalosporin-resistant *Enterobacterales*, as a proxy of underlying resistance mechanisms, such as extended-spectrum beta-lactamases (ESBL) and AmpC beta-lactamases. Considering that the presence and expressions of these enzymes differ among distinct *Enterobacterales* isolates, and the hydrolytic profile of each enzyme may impact on different therapeutic approaches, the panel analyzed each of these mechanisms in specific sections.

One panelist was selected for literature review and first draft of the recommendations. The MDR-GNB for each panelist review was grouped as follow: 1) CRE, 2) CRAB, 3) carbapenem-resistant *P.* a*eruginosa* (CRPA), 4) ESBL and AmpC-producing *Enterobacterales*, and 5) *S. maltophilia* and *B. cepacia*. The panel meetings were performed online. The initial recommendations were presented in a dedicated section during the 23rd Brazilian Congress of Infectious Diseases. After this presentation, and considering the inputs provided by the audience, there was a presential meeting. All remaining meetings were performed online. The revised version of the draft was also submitted to public consultation at the Brazilian Society of Infectious Diseases website during August 2024. A revised draft was elaborated by two panelists and presented to final inputs and approval of all members of the panel.

### Structure of recommendations

With the exception of *S. maltophilia* and *B. cepacia*, each pathogen-resistance section was divided in respiratory tract infections (RTIs), skin and soft tissue infections (SSTIs), primary bloodstream infections (PBSIs) and intra-abdominal infection (IIAs). For better readability, these infection sites may occasionally be referred to as “systemic infections”, acknowledging that acute pyelonephritis (APN) can also be considered a “systemic” infection. However, because of some particularities of the urinary tract, APN was presented separately, in the same section of complicated lower urinary tract infections (cLUTIs). cLUTIs were defined as infections occurring in the presence of factors that impair urinary tract function or host defenses. These factors include urinary obstruction, neurogenic urinary retention, immunosuppression, renal failure, renal transplantation, pregnancy, male sex, and the presence of foreign bodies, indwelling catheters, or other drainage devices.^13^^,^^14^ Uncomplicated (uLUTI) was also presented in an additional section.

Considering the paucity of evidence for providing recommendations for each bacteria-resistance separately in the treatment of difficult-to-treat sites of infections, such as central nervous system (CNS), endocarditis and osteomyelitis, a section presenting some general suggestions in the management of these infections was provided after bacteria-resistance-specific sections.

### How were antimicrobial therapies selected for recommendations?

No new systematic review was performed for the elaboration of this guideline. The panel considered the reviews performed in guidelines published in the last five years.^4^, ^5^, ^6^, ^7^, ^8^, ^9^, ^10^, ^11^ Overall, supporting evidence for this guideline’s recommendations can be found in previously published ones.^4^, ^5^, ^6^, ^7^, ^8^, ^9^, ^10^, ^11^ Studies published after the latest published guideline were also considered by the panel for recommendations if deemed appropriate. Whenever a recommendation of a specific antimicrobial was in agreement to previously published guidelines,^4^, ^5^, ^6^, ^7^, ^8^, ^9^, ^10^, ^11^ no additional reference was cited in the text.

Recommendations were preferably based on clinical studies. However, the quality of studies was also taken into account in the decision process. *In vitro*, Pharmacokinetics (PK) and PK/pharmacodynamics (PD), and experimental and *in vitro* infection models were also analyzed, and weighted accordingly in the recommendations. Whenever no clinical evidence exists to support a given drug or therapeutic strategy, expert opinions of members of the panel can be identified by “the panel recommends/does not recommend or suggests/does not suggest”.

## Questions and recommendations

### General issues

#### How should the recommendations be interpreted?

This guideline provides recommendations for target therapy of infections caused by the most frequent MDR-GNB in the hospital settings, and must be interpreted as general recommendations that may be individualized for some specific patients, and according to the availability of the antimicrobials in each setting.

The guideline does not discuss the different criteria for any of the infection sites addressed in the recommendations. Hence, a careful assessment should be performed to ensure that the microorganism recovered in cultures is indeed the cause of the infection and not a colonizer.

Empiric treatment decisions are beyond the scope of this document, and as general principles the choice of the best empiric therapy should be based on: i) previous organisms identified from the patient and associated antimicrobial susceptibility testing (AST) data; ii) previous antibiotic exposure during current or recent hospitalizations; and iii) local AST patterns for the most likely bacteria according the site of infection, unit of infection and, occasionally, locally identified risk factors for acquisition of a MDR-GNB.

##### Selection of a specific agent

In general, if a given pathogen is susceptible to a narrower-spectrum antimicrobial agent, and this agent is appropriate for the specific infectious syndrome/site, preference should be given to its use. This approach minimizes the secondary and undesirable impact on the patient's natural microbiota and further selection pressure for bacteria with resistance to broader spectrum agents. In addition, when two antibiotics exhibit comparable efficacy, key factors in choosing a particular drug include safety profile, cost-effectiveness, ease of use, and availability in the local formulary.

##### Treatment duration

This document does not specify treatment durations, as they should be tailored to individual cases based on scientific evidence, which generally favors the shortest effective course.^15^, ^16^, ^17^, ^18^ Effective infection source control, such as abscess drainage and catheter removal, is essential for minimizing treatment duration and ensuring optimal infection resolution. Importantly, the recommendations provided in this document should not replace clinical judgment in specific situations not covered by this guideline, where individual patient characteristics must be carefully considered.

#### How should antimicrobial susceptibility tests be interpreted in clinical practice?

Since 2019, Brazilian laboratories have adopted the recommendations of the Brazilian Committee on Antimicrobial Susceptibility Testing (BrCAST),^19^ a national committee of the European Committee on Antimicrobial Susceptibility Testing (EUCAST), in accordance with Ordinance nº 64 of 12/11/2018.^20^ In addition, for antimicrobials to which there is no breakpoint available, we defined the conditions in which they may be considered as having *in vitro* activity (Table 1).

**Table 1:** Classification of antimicrobials concerning their *in vitro* activity for this guideline.

**Antimicrobials with *in vitro* activity:**	**Antimicrobial**	**Gram-negative bacilli**
Category (S): “S within parentheses” means that there are no breakpoints for monotherapy use in the AST a	Polymyxin B or colistin MIC ≤2 mg/L	*A. baumannii, Enterobacterales* and *P. aeruginosa*
	Aminoglycosides (S) for systemic infections	*A. baumannii, Enterobacterales* and *P. aeruginosa*^b^
Antimicrobials without criteria for interpretation of AST	Tigecycline MIC ≤0.5 mg/L^c^	*A. baumannii, Enterobacterales*^d^ and *S. maltophilia.*
	Ampicillin-sulbactam MIC ≤8/4 mg/L^c^	*A. baumannii*^e^
	Aztreonam-avibactam ≤4/4 mg/L	*Enterobacterales, P. aeruginosa,* and *S. maltophilia*^f^
	TMP-SMX MIC ≤2 mg/L^g^	*S. maltophilia* and *B. cepacia*
	Levofloxacin MIC ≤0.5 mg/L^c^	*S. maltophilia* and *B. cepacia*
	Ceftazidime ≤4 mg/L^c^^,^^h^	*B. cepacia*
	Ceftazidime-avibactam ≤8/4 mg/L^h^	*B. cepacia*
	Meropenem ≤2 mg/L^c^	*B. cepacia*
Antimicrobials categorized as Susceptible (S) or Susceptible Increased Exposure (I) in AST	All with AST breakpoint i	*A. baumannii, Enterobacterales, P. aeruginosa* and *S. maltophilia*
Antimicrobials with potential for *in vivo* activity through optimized dosing regimens^j^		
	Ampicillin-sulbactam MICs of 16/8 to 64/32 mg/mL	*A. baumannii*^e^
	Meropenem MICs of 16 or 32 mg/L	*A. baumannii, Enterobacterales*, and *P. aeruginosa*
	Tigecycline MICs of 1 or 2 mg/L	*A. baumannii* and *Enterobacterales*
	Aztreonam-avibactam ≤16/4 mg/L	*P. aeruginosa* and *S. maltophilia*^k^

AST, antimicrobial susceptibility test; MIC, minimal inhibitory concentration, TMP-SMX, trimethoprim-sulfamethoxazole.

a According to the BrCAST/EUCAST breakpoints.

b Note that the doses currently recommended for the amikacin breakpoint by BrCAST/EUCAST for all Gram-negative bacilli are not those recommended in this guideline. Gentamicin has no longer breakpoints for *P. aeruginosa*.

c Based on the pharmacokinetics/pharmacodynamics (PK/PD) breakpoints from previous BrCAST documents and the document “When there are no breakpoints in breakpoint tables?” version 2023-06-30.).

d Tigecycline breakpoints are only established for *Escherichia coli* and *Citrobacter freundii. Morganella morganii, Serratia marcescens, Proteus* spp., and *Providencia* spp. are intrinsically resistant to tigecycline. For other *Enterobacterales*, consider tigecycline as having *in vitro* activity if MIC ≤0.5 mg/L.

e For *A. baumannii* the active component is sulbactam MIC = 4 mg/mL.

f Based on *Enterobacterales* susceptibility breakpoint.

g Breakpoint for *S. maltophilia*, also suggested for considering *in vitro* activity for *B. cepacia*. Breakpoint based on the trimethoprim concentration in an 1:19 trimethoprim:sulfamethoxazole ratio.

h The susceptibility breakpoint for *P. aeruginosa* is 8 mg/L for ceftazidime.

i The “I” category for meropenem should not be interpreted as susceptible to increased exposure, particularly if the isolate produces a carbapenemase. In these situations, meropenem should usually be a part of a combination scheme with another *in vitro* active antimicrobial.

j Potential *in vivo* activity means that a substantial proportion of patients (≥50 %) may attain the PK/PD target associated with maximal bacterial killing or at least a static effect, using optimized drug posology.

k Based on *P. aeruginosa* susceptibility breakpoint for aztreonam alone.

In 2019, EUCAST modified the definition of “Intermediate” category, meaning “Susceptible, Increased Exposure”, while the symbol “I” was retained. This category actually indicates that the susceptibility breakpoints apply to the highest doses of the antimicrobials. This is particularly important in *P. aeruginosa* and *S. maltophilia*, in which the former “S” breakpoints values were just changed by the symbol “I”. It means that for almost all antimicrobials, the “I” should be literally read as “S”, provided that the recommended dosing regimen is followed (see Table 2). A single exception should be made for carbapenems, since the current “I” is applied to the same minimal inhibitory concentration (MIC) values (for example, 4 and 8 mg/L of meropenem) of the pre-2019 “I” category. Hence, carbapenems are the single exception in which there is no evidence supporting the use of these drugs in monotherapy for treatment of carbapenemase-producing GNB infections by isolates categorized as “I”, or even “S”.^4^, ^5^, ^6^, ^7^, ^8^, ^9^, ^10^, ^11^

**Table 2:** Suggested antimicrobial dosing for the treatment of carbapenem-resistant Gram-negative bacilli infections in adults, assuming normal renal and hepatic function.

Antimicrobials	Dosage	Potential use for
Beta-lactams and beta-lactam-beta-lactamase inhibitors		
Ampicillin-sulbactam	Administer a total daily dose of 9–12 g of sulbactam through one of the following regimens: 9 g ampicillin-sulbactam (6 g ampicillin, 3 g sulbactam) IV, every 6–8 h, over 4 h, or 27 g ampicillin-sulbactam (18 g ampicillin, 9 g sulbactam) IV, as a continuous infusion over 24 h	CRAB
Cefepime	Uncomplicated cystitis: 2 g IV every 8 h, infused over 30-min	AmpC-producing *Enterobacterales*
	Other infections: 2 g IV every 8 h, infused over 3-h	
Ceftazidime	2 g IV every 8 h, infused over 30-min for specific ESBL-producing *Enterobacterales* (see recommendations)	ESBL-producing *Enterobacterales;B. cepacia*
	2 g IV every 8 h, infused over 3 h for non-carbapenemase-producing carbapenem-resistant *P. aeruginosa* as a part of combination therapy if *in vitro* activity (see recommendations).	
Ceftazidime-avibactam	2.5 g IV every 8 h, infused over 3-h	Class A-producing CRE, Class A- and non-carbapenemase-producing CRPA, *B. cepacia.*
Ceftazidime-avibactam plus aztreonam	Ceftazidime-avibactam: 2.5 g IV every 8 h, infused over 3-h plus aztreonam 2 g IV every 8 h infused over 3-h (administered simultaneously)	Class B-producing CRE, Class B-producing CRPA, *S. maltophilia*
Ceftolozane-tazobactam	Uncomplicated cystitis: 1.5 g IV every 8 h, infused in 1-h	Non-carbapenemase-producing CRPA
	Other infections: 3 g IV every 8 h, infused over 3-h	
Imipenem-cilastatin- relebactam	1.25 g IV every 6 h, infused in 30-min	Class A-producing CRE, Class A- and non-carbapenemase-producing CRPA
Meropenem	Usual: 1 g in 30-min every 8 h.	Usual: AmpC-producing *Enterobacterales;* ESBL-producing *Enterobacterales*
	High: 2 g infused over 3-h every 8 h	High: CRE, CRPA, CRAB as a part of combination schemes; *B. cepacia.*
Piperacillin-tazobactam	4.5 g infused over 3-h every 6 h	ESBL-producing *Enterobacterales*
Monobactam		
Aztreonam	2 g infused over 2‒3 h every 8-h	Class B-producing CRE; Class B-producing CRPA
Polymyxins		CRE, CRPA, CRAB
Colistin	Loading dose (regardless of the severity of the infection, or glomerular filtration rate): 300 mg colistin (9000,000 IU colistimethate) in 1‒2 h. Subsequent doses, start 12 h to 24 h after loading dose: glomerular filtration rate >60 mL/min: 300 mg colistin (9000,000 IU colistimethate) / day administered every 8 h or 12 h.	
Polymyxin B	2.5‒3.0 mg or 25,000‒30,000 IU/kg/day administered every 12 h: Loading dose for severe infections: 2.0 mg or 20,000 IU/kg. We do not recommed single dose over 200 mg or 2,000,000 IU.	
Fluoroquinolones		
Ciprofloxacin	Uncomplicated cystitis: 400 mg IV every 12 h or 500 mg PO every 12 h	CRE, CRPA, CRAB
	Other infections or infections at any site by *P. aeruginosa:* 400 mg IV every 8 h or 750 mg PO every 12 h	
Levofloxacin	750 mg IV or PO once daily	*S. maltophilia, B. cepacia*
Glicylcyclines		
Tigecycline	Loading dose: 200 mg IV followed by 100 mg IV every 12 h	CRE, CRAB
Aminoglycosides		
Amikacin	Uncomplicated cystitis: 15 mg/kg IV once daily	AmpC-producing *Enterobacterales*, ESBL-producing *Enterobacterales*, CRE, CRPA, CRAB
	Pyelonephritis or complicated lower urinary tract infections: 15 mg/kg IV once daily.	
	Other Infections: 20 mg/kg IV once daily.	
Gentamicin	Uncomplicated cystitis: 5 mg/kg/dose IV once daily.	AmpC-producing *Enterobacterales*, ESBL-producing *Enterobacterales*, CRE
	Pyelonephritis or complicated lower urinary tract infections: 7 mg/kg IV once daily.	
Tobramycin	Uncomplicated cystitis: 5 mg/kg IV dose once daily.	AmpC-producing *Enterobacterales*, ESBL-producing *Enterobacterales*, CRE, CRPA, CRAB
	Pyelonephritis or complicated lower urinary tract infections: 7 mg/kg IV once daily	
Other drugs		
Trimethoprim- Sulfamethoxazole (TMP-SMX)	Uncomplicated cystitis: 160/800 mg (TMP/SMX) IV/PO every12 h	AmpC-producing *Enterobacterales*, ESBL-producing *Enterobacterales*, CRE, CRAB, *S. maltophilia, B. cepacia*
	Other infections: 10‒15 mg/kg/day (TMP component) IV or PO divided every 6 h to 12 h	
Fosfomycin	Uncomplicated cystitis: 3 g PO once daily	*E. coli*
Nitrofurantoin	Uncomplicated cystitis: 100 mg PO every 6 h	*E. coli*

IV, intravenous; PO, oral route; h, hours; mg/kg, milligrams per kilogram; CRAB, carbapenem-resistant *Acinetobacter Baumannii*; CRE, carbapenem-resistant *Enterobacterales*; CRPA, carbapenem-resistant *P.* a*eruginosa*; ESBL, extended-spectrum beta-lactamases.

BrCAST/EUCAST breakpoints also have antimicrobials categorized as (S), “S in parentheses”, which means that there are no breakpoints for monotherapy use of a given antimicrobial. There are also some antimicrobials without species-specific breakpoints, as presented ahead. In these circumstances MICs of antimicrobial agents were considered based on their potential for *in vivo* rescue of activity through the optimization of dosing regimens as shown in Table 1, which are generally in accordance to the “EUCAST guidance on When there are no breakpoints in breakpoint tables?” document.^21^

### Resistance mechanisms

#### What are the major resistance mechanisms determining resistance to antimicrobial agents addressed in this document?

##### Carbapenem-resistant *Enterobacterales*

Although production of ESBL and/or AmpC beta-lactamases coupled with porin modification have been associated with low levels of carbapenem resistance, the production of carbapenemases has been the main mechanism reported among Brazilian *Enterobacterales* isolates. KPC (class A carbapenemase) has been detected as the most frequent carbapenemase, but an increasing frequency of NDM (class B, metallo-beta-lactamases) has been observed in distinct Brazilian hospitals.^2^^,^^22^ An increase in the co-production of class A and class B carbapenemases have also been increasingly reported since the COVID-19 pandemic in Brazilian hospitals.^2^^,^^22^ Although less frequent, other class A and B carbapenemases have been reported in Brazil.^23^ The OXA-370, a variant of OXA-48 is the main class D carbapenemase reported in *Enterobacterales* recovered in Brazilian hospitals.^24^

##### Carbapenem-resistant A*cinetobacter baumannii*

Class D carbapenemase, particularly OXA-23, is the main mechanism determining resistance to carbapenems worldwide, including in Brazil.^25^^,^^26^ Other class D carbapenemases, such as OXA-58, −72 −143,−231, −253 have also been reported.^25^^,^^26^ Although less common, class B carbapenemases such as IMP-, VIM- and NDM- types have also been described.^2^^,^^25^^,^^26^

##### Carbapenem-resistant P*seudomonas aeruginosa*

Carbapenem resistance may result from the co-expression of multiple mechanisms, including decreased outer membrane permeability (such as alterations in outer membrane proteins like OprD), overexpression of efflux pumps (particularly MexAB-OprM), and AmpC hyperproduction, and/or carbapenemase production.^27^ Carbapenemase production, mainly class B, is also a mechanism driving resistance to carbapenems in *P. aeruginosa* isolates, although this pathogen may be resistant to carbapenems through a combination of multiple mechanisms, such as AmpC derepression, hyperexpression of efflux pumps and decreased expression or modifications in OprD porin. SPM-1 used to be the most common class B carbapenemase in *P. aeruginosa*.^27^ However, recent studies demonstrated that other metallo-beta-lactamases, particularly NDM, have replaced SPM-1 as the major carbapenemase in Brazil.^2^^,^^22^ A significant proportion of CRPA do not produce carbapenemase but exhibit alternative resistance mechanisms, such as overexpression of efflux pumps and alterations in the OprD porin.^2^^,^^22^ Although the European Society of Clinical Microbiology and Infectious Diseases (ESCMID) and the Infectious Diseases Society of America (IDSA) guidelines document refer to the classification of “difficult-to-treat resistance ”,^4^^,^^5^ in this document we have chosen to use only the WHO classification, CRPA based on criteria adopted for interpreting antimicrobial susceptibility results in Brazil.^12^

##### Important resistances in *S. maltophilia*

*S. maltophilia* is a non-glucose-fermenting, GNB with ubiquitous distribution, commonly found in moist environments. It produces two beta-lactamases: L1, a class B metallo-beta-lactamase (carbapenemase), and L2, a a serine-beta-lactamase ‒ that confer intrinsic resistance to beta-lactam antibiotics, including penicillins, cephalosporins and aztreonam. The species also exhibits intrinsic resistance to aminoglycosides and tetracyclines. Additionally, *S. maltophilia* isolates often harbor resistance determinants such as *Smqnr*, which impair fluoroquinolone binding to topoisomerases, and it commonly overexpresses efflux pumps, further reducing antimicrobial susceptibility.^28^ The main mechanism of TMP-SMX resistance in *S. maltophilia* has been reported as acquisition of the *sul1, sul2,* and *drfA* genes.^29^

##### Important resistances in *Burkholderia cepacia* complex

*Burkholderia cepacia* complex (Bcc) bacilli are widely distributed in the environment, particularly in humid conditions. Their resistance mechanisms are multifactorial, involving reduced membrane permeability, active efflux systems, and enzymatic antibiotic degradation, making infections caused by these bacteria especially difficult to treat.^30^ Resistance to beta-lactam antibiotics within the Bcc is primarily mediated by class A beta-lactamases, notably PenB, and AmpC. *B. cenocepacia* and *B. multivorans* also harbor PenA. Overexpression of these enzymes significantly reduces susceptibility to beta-lactams such as ceftazidime and meropenem. In *B. cenocepacia*, six RND efflux systems have been identified. Among these, RND-3, RND-4, and RND-10 play key roles in antimicrobial resistance. Specifically, RND-3 and RND-4 contribute to aminoglycoside resistance; RND-4 mediates resistance to azithromycin; both RND-4 and RND-10 are involved in chloramphenicol resistance; and all three systems (RND-3, RND-4, and RND-10) contribute to resistance against fluoroquinolones and tetracyclines. Additionally, RND-10 is associated with resistance to trimethoprim.^31^ Additionally, target site modifications are mainly responsible for resistance to fluoroquinolones and trimethoprim, while resistance to polymyxins is partly due to a unique lipopolysaccharide (LPS) structure that hinders polymyxin binding to the bacterial outer membrane.^30^

##### Defining extended-spectrum beta-lactamase-producing *enterobacterales* and AmpC-producing E*nterobacterales* and key points on antimicrobial susceptibility tests

ESBLs belong to molecular class A according to the Ambler classification. These enzymes are capable of hydrolyzing penicillins, monobactams, broad-spectrum cephalosporins, including third- and fourth-generation agents, as well as newer cephalosporins such as ceftaroline and ceftolozane.^32^ ESBLs activity are *in vitro* inhibited by both classical beta-lactamase inhibitors (clavulanic acid, sulbactam, and tazobactam) and newer agents such as avibactam, relebactam, and vaborbactam. Although ESBLs are most frequently reported in *Enterobacterales*, particularly in species such as *K. pneumoniae, Escherichia coli*, and *Proteus mirabilis*, the genes encoding these enzymes can also be found in other bacterial species, as they are typically located on plasmids that can be transferred between bacteria. In this document, third-generation cephalosporin-resistant *K. pneumoniae, E. coli* and *P. mirabilis* will be considered ESBL-producing *Enterobacterales* (ESBL-E)*.* AmpC beta-lactamases (AmpC) belong to Ambler class C. Similar to ESBLs, they hydrolyze all penicillins, monobactams, and broad-spectrum cephalosporins. AmpC enzymes are inhibited only by novel beta-lactamase inhibitors, but exhibit a low hydrolysis rate for cefepime.^33^ AmpC-encoding genes are chromosomally located in several *Enterobacterales* species, including *Enterobacter cloacae, Klebsiella aerogenes, Citrobacter freundii, Serratia marcescens, Morganella morganii*, and *Providencia stuartii*. In contrast to ESBLs, exposure to beta-lactams can trigger AmpC hyperproduction, either through inducible expression or selection of derepressed mutants.^33^^,^^34^ This may result in the emergence of resistance during treatment and subsequent therapeutic failure, even when an isolate initially tests as susceptible *in vitro*. Although it is recognized that the risk of therapeutic failure with third-generation cephalosporins due to AmpC induction is higher in certain species, such as *E. cloacae, K. aerogenes*, and *C. freundii*, clinical evidence remains limited to definitively support the safe use of third-generation cephalosporins in infections caused by other species, such as *S. marcescens, M. morganii*, and *P. stuartii*.^33^^,^^34^

#### When the characterization of resistance mechanisms associated with an antimicrobial-resistant profile is recommended?

Carbapenemase detection tests may imply additional costs in the routine practice of clinical laboratories. However, the implementation of rapid tests (phenotypic, immunochromatographic or PCR) for carbapenemases detection may provide a first guide to the choice of empirical therapy while awaiting the results of ASTs.

We recommend performing rapid phenotypic tests, such as Carba NP and Blue Carba.^35^ Isolates of carbapenem-resistant *Enterobacterales* and carbapenem-resistant *P. aeruginosa* that are phenotypically identified as carbapenemase producers by these tests can subsequently be submitted for confirmation of the carbapenemase class, preferably using immunochromatographic assays and/or PCR. Phenotypic tests, such as Carba NP or Blue Carba combined with EDTA or newer beta-lactamase inhibitors, can also be employed. Alternatively, other phenotypic methods like CIM and mCIM may be used.^35^, ^36^, ^37^ While these tests generally demonstrate high sensitivity and specificity, they do have limitations. Therefore, understanding the local epidemiology, both in terms of prevalent pathogens and the enzymes of interest, is essential to select the most appropriate testing method.

### Antimicrobial doses

#### What are the doses recommended for the treatment of MDR-GNB?

The suggested dosing regimens for the treatment of MDR-GNB infections are based on the specific characteristics of the targeted MDR-GNBs and are presented in Table 2.

### Carbapenem-resistant *Enterobacterales*

#### What are the recommendations for treatment of RTI, SSTI, PBSI and IAI by class A carbapenemase-producing CRE?

Beta-lactam/beta-lactamase inhibitors (BL/BLIs) combinations, either ceftazidime-avibactam or imipenem-relebactam, are the preferred options for the treatment of systemic infections by class A carbapenemase-producing CRE as shown in Table 3. There is no direct comparison between these two agents in terms of clinical efficacy for CRE. There may be resistance to one of these options and susceptibility to the other.^38^ Imipenem-relebactam has lower *in vitro* activity than ceftazidime-avibactam against *Enterobacterales* of the *Morganellaceae* family (*Morganell*a spp., *Proteus* spp. and *Providencia* spp.); therefore, ceftazidime-avibactam should be preferred for the treatment of species of the *Morganellaceae* family.^19^

**Table 3:** Recommendations for the treatment of class A, B, D, and non-carbapenemase producing (NC) carbapenem-resistant *Enterobacterales* (CRE).

Infection site	Treatment options (Carbapenemase type: A/D/NC, B or ANY type)	
LRT, SSTI, PBSI, IAA	**Preferential**	**Alternative**
	Ceftazidime-avibactam (A/D/NC) or imipenem-relebactam (A/NC)^a^^,^^b^	Polymyxin B or colistin combined with meropenem, tigecycline, or an aminoglycoside (ANY)^e^^,^^f^
		Ciprofloxacin, levofloxacin (ANY)^g^
	Ceftazidime-avibactam plus aztreonam (B)^c^^,^^d^	TMP-SMX (ANY)^g^
		Imipenem-cilastatin-relebactam plus aztreonam (B)^h^^,^^i^
APN, cLUTI		
	Ceftazidime-avibactam (A/D/NC) or imipenem-cilastatin-relebactam (A/NC)^a^^,^^b^	Amikacin, gentamicin, tobramycin (ANY)^l^
	Ceftazidime-avibactam plus aztreonam (B)^c^^,^^d^	Polymyxin B or colistin (ANY)^m^
	Ciprofloxacin, levofloxacin, TMP-SMX (ANY)^j^	Imipenem-cilastatin-relebactam plus aztreonam (B)^h^^,^^i^
	Aztreonam (B)^k^	Tigecycline (ANY)^n^
uLUTI		
	Fosfomycin, Nitrofurantoin (ANY)^o^	Amikacin, gentamicin, tobramycin (ANY)
	Ciprofloxacin, levofloxacin, TMP-SMX (ANY)^p^	Polymyxin B or colistin (ANY)^m^
	Ceftazidime-avibactam (A/D/NC) or imipenem-cilastatin-relebactam (A/NC)^a^^,^^b^^,^^q^	Imipenem-cilastatin-relebactam plus aztreonam (B)^h^^,^^i^^,^
	Ceftazidime-avibactam plus aztreonam (B)^c^^,^^d^^q^	Tigecycline (ANY)
	Aztreonam	

APN, acute pyelonephritis; cLUTI, complicated urinary tract infection; LRTI, lower respiratory tract infection; AI, intra-abdominal infection; PBSI, primary bloodstream Infection; SSTI, skin and soft tissue infection; TMP-SMX, trimethoprim-sulfamethoxazole; uLUTI; uncomplicated LUTI.

a Imipenem-cilastatin-relebactam has lower *in vitro* activity than ceftazidime-avibactam against *Enterobacterales* of the *Morganellaceae* family (*Morganella* spp., *Proteus* spp. and *Providencia* spp.). For other *Enterobacterales* species, there is no evidence to support the preferential use of one antimicrobial agent over the other. The panel suggests that ceftazidime-avibactam should be preferred due to its narrower spectrum. No data is supporting the use of these agents in combination with other antimicrobials from different classes; both should be used as monotherapy.

b Only ceftazidime-avibactam has activity against OXA-48-producing isolates (OXA-48 is a class D carbapenemase). In Brazil, OXA-370 is the most frequently detected OXA-48 variant.

c Ceftazidime-avibactam has no activity against class B-producing isolates. Aztreonam is not hydrolyzed by class B, but can be hydrolyzed by other beta-lactamases produced by the bacteria. The combination of ceftazidime-avibactam with aztreonam may restore *in vitro* activity of aztreonam against class B carbapenemase-producing CRE. Aztreonam should be infused simultaneously with ceftazidime-avibactam for best efficacy.

d Susceptibility to aztreonam-avibactam can be tested by disk diffusion or gradient strips. Synergism of this combination can be tested by the disk pre-diffusion technique or disk elution, but it does not substitute the results of susceptibility test.

e We suggest that a polymyxin be used in combination with a second antimicrobial agent, preferably categorized as S, I or (S), in that order of preference. If none are available, an antimicrobial with the possibility of rescuing the action *in vivo*, such as meropenem or tigecycline, if any of their respective minimal inhibitory concentrations (MICs) are within the range presented in Table 1. Aminoglycosides should be prescribed last due to their greater potential for nephrotoxicity. If no antimicrobials fall into these categories, combination therapy is still recommended; however, there is no evidence to recommend one antimicrobial over another. If MIC determination is not available, we suggest combination therapy with meropenem.

f In the presence of confirmed *in vitro* resistance to a polymyxin, and in the lack of access to both ceftazidime-avibactam and imipenem-cilastatin-relebactam, a combination of two drugs categorized with at least one categorized as S, I or (S), or with the possibility of rescuing *in vivo* activity, in that order of preference, should be pursued. The choice of the second agent should follow the same order. If none are available, we recommend the combination of meropenem with tigecycline. If *in vitro* resistance is present, we do not recommend the use of polymyxins, aminoglycosides, fluoroquinolones, or TMP-SMX, owing to the narrow therapeutic window which discourage the use of doses higher than those described in Table 2, associated with the lack of evidence that antimicrobial activity may be rescued in isolates categorized as R for these drugs.

g If categorized as susceptible, either a fluoroquinolone or TMP-SMX should be part of a combination scheme; however, this phenotype is uncommon in CRE isolates. In selected cases, in non-critically ill patients, or in less severe infections, monotherapy with fluoroquinolone or TMP-SMX may be considered.

h Although imipenem-cilastatin-relebactam also restores the activity of aztreonam against class B carbapenemase-producing isolates, this is not the preferred option because the activity of this combination has been demonstrated in fewer isolates than ceftazidime-avibactam, and clinical data on the efficacy and safety of imipenem-cilastatin-relebactam plus aztreonam are lacking.

i The combination of aztreonam with relebactam is not commercially available for testing by disk diffusion or gradient strips. Furthermore, synergism cannot be extrapolated from the combination of aztreonam with avibactam, nor from antimicrobial susceptibility testing results for aztreonam–avibactam.

j If categorized as susceptible, either a fluoroquinolone or TMP-SMX should be considered as first-line agents, particularly in cLUTI, in non-critically ill patients.

k If susceptible, aztreonam may be used in monotherapy in non-severe APN, cUTI, or as a part of combination scheme for more severe cases.

l When the potential for nephrotoxicity is considered acceptable, aminoglycosides are second-line options in stable, non-critically ill patients. For APN or in critically ill patients, we recommend that aminoglycosides should be used in combination with a second antimicrobial agent preferably categorized as S, I or (S), in that order, or with the possibility of rescuing the *in vivo* action, such as meropenem, if they present with MICs described above. Polymyxins and aminoglycosides are the last preferred combination because of the greatest potential for nephrotoxicity.

m For APN, cLUTI, and uLUTI, colistin is the preferential option, because of higher urinary concentrations compared to polymyxin B. Combination therapy is recommended for APN and in cLUTI in critically ill patients. The options for combining are those presented in footnote “e”, with the difference that aminoglycosides are preferred over tigecycline, unless the possibility of any degree of acute kidney injury is unacceptable for the patient.

n Tigecycline has low urinary concentrations and should not be among the first options. Only use as monotherapy if presenting *in vitro* activity and no other option is available.

o Both are options only for *E. coli*.

p If categorized as susceptible, either a fluoroquinolone or TMP-SMX should be considered as first-line agents, against non-*E. coli* isolates.

q Consider sparing these antimicrobials for more severe infections, use only if the isolates are resistant to previous options.

The panel suggests that ceftazidime-avibactam may be preferred due to its narrower antimicrobial spectrum. Clinical studies could not find benefit in adding a second agent to ceftazidime-avibactam in the treatment of KPC-producing CRE.^39^ Although there is no comparative study with imipenem-relebactam, there is *a priori* no reason to expect that the addition of a second antimicrobial will improve clinical efficacy of imipenem-relebactam either. Therefore, monotherapy with one of these BL/BLIs combinations is recommended.

If there is susceptibility to other non-beta-lactam antimicrobial, such as fluoroquinolones or TMP-SMX, these drugs may be considered as monotherapy in an individual basis, since there is no clinical studies evaluating the use of these antimicrobials in systemic infections by class A carbapenemase-producing CRE.

Polymyxins in combination with meropenem, tigecycline or aminoglycosides are the preferred alternative regime, when the BL/BLIs are not available. There is no recommendation for the use of any of these agents in monotherapy for systemic infections. The panel suggests that the choice of combination should be based on the susceptibility profile: if the isolates present susceptibility to fluoroquinolones or TMP-SMX, or susceptibility increasing exposure (“I”) to meropenem, one of these agents should be preferentially used in combination with a polymyxin. If the isolate does not present Susceptibility (S or I) to any other agent, the preferred combination is the agent that presents the possibility of PK/PD rescue of activity as presented in Table 1. If more than one agent presents the possibility of rescue of activity through PK/PD optimization, the choice should rely on the availability of the drug, the site of infection and potential toxicities of each option. Although some isolates may present susceptibility within brackets to aminoglycosides, the panel recommends their use in combination with polymyxins in systemic infections should be regarded as the last option due to their higher potential for nephrotoxicity. However, this decision should be individualized, and aminoglycosides may be considered as a combination option, particularly when MIC data for other antimicrobials are unavailable.

#### What are the recommendations for treatment of APN or cLUTI by class A carbapenemase-producing CRE?

The recommendations for the treatment of APN and cLUTI is similar to those for RTI, SSTI, primary BSI and IAI with the difference that other non-beta-lactam antimicrobial, such as fluoroquinolones or TMP-SMX can be used in monotherapy as preferred agents if there is *in vitro* susceptibility to these drugs. In addition, as alternative agents, aminoglycosides or polymyxins (preferably, colistin) may be considered in monotherapy for complicated but non-severe cystitis, when the potential for nephrotoxicity is considered acceptable. For more severe cLUTI or APN, the panel suggests that these drugs be used in combination with a second antimicrobial agent preferably categorized as S, I or (S), in that order, or with the possibility of rescuing the *in vivo* action, such as meropenem. Due to its low concentration in the urinary tract, tigecycline should be regarded as the last option for composing a combination scheme.

#### What are the recommendations for treatment of RTI, SSTI, PBSI and IAI by class B carbapenemase-producing CRE?

Ceftazidime-avibactam in combination with aztreonam is the preferred option for the treatment of systemic infections by class B carbapenemase-producing CRE.^40^ Although in theory aztreonam could be combined with other novel BL/BLI, *in vitro* studies have shown overall superior activity of aztreonam combined with avibactam compared to relebactam and vaborbactam against class B carbapenemase-producing CRE.^41^, ^42^, ^43^, ^44^ In addition, to date, no clinical study reported the use of aztreonam in combination with imipenem-relebactam for the treatment of CRE.

Notably, in some isolates aztreonam alone may present *in vitro* activity. In these cases, the panel recommends that aztreonam may be the preferential agent for combination therapy with a polymyxin. As monotherapy, aztreonam may only be considered for stable patients with no severe infections.

The use of other non-BL/BLIs antimicrobials should be considered in the same manner as for class A carbapenemase-producing CRE.

#### What are the recommendations for treatment of APN or cLUTI by class B carbapenemase-producing CRE?

The recommendations for the treatment of APN and cLUTI are similar to those for class A carbapenemase-producing CRE, with the exception that ceftazidime-avibactam in combination with aztreonam should be used instead of ceftazidime-avibactam or imipenem-relebactam-alone. If susceptible, the panel suggests that aztreonam in monotherapy can be used for cLUTI, and for APN in selected stable patients. It may also be the preferred agent for combination therapy for APN or cUTIs in more severe infections.

#### What are the recommendations for treatment of uLUTI by CRE?

TMP-SMX or a fluoroquinolone are the preferred agents for the treatment of uLUTI regardless of the mechanisms of resistance to carbapenems, if there is *in vitro* susceptibility. Fosfomycin and nitrofurantoin are antimicrobials that can be used in uLUTI caused by carbapenem-resistant *E. coli*. Aminoglycosides may be used in monotherapy for uLUTI by CRE, regardless of the mechanisms of resistance to carbapenems, if there is *in vitro* susceptibility. Aztreonam in monotherapy may be used for class B carbapenemase-producing CRE.

#### What are the recommendations for antimicrobial therapy of class D carbapenemase-producing CRE?

The recommendations for treating infections caused by CRE producers of OXA-48 or its variants (class D carbapenemases) are generally the same as for class A enzymes across all body sites, with the exception that imipenem-relebactam is not an option. When *in vitro* susceptibility is confirmed, ceftazidime-avibactam remains the only recently developed BL/BLIs with reliable activity against these pathogens. This is largely because ceftazidime is not efficiently hydrolyzed by OXA-48.^4^, ^5^, ^6^, ^7^, ^8^, ^9^

#### What are the recommendations for antimicrobial therapy of non-carbapenemase-producing CRE?

The recommendations for the treatment of non-carbapenemase-producing CRE are the same as those for class A carbapenemase-producing CRE.

### Carbapenem-resistant *Acinetobacter baumannii*

#### What are the recommendations for treatment of RTI, SSTI, primary BSI and IAI by CRAB?

The panel recommends the use of a combination of two antimicrobials with *in vitro* activity (Table 4). Considering the BrCAST/EUCAST breakpoints for CRAB, only ciprofloxacin and TMP-SMX could be a combination of two antimicrobials with the “S” phenotype. However, this profile is rarely present in CRAB isolates, and clinical studies supporting their use in systemic infections by CRAB are scanty. Therefore, the combination regimen will necessarily include antimicrobials with susceptibility within brackets (for definitions, see section 1.2 of this guideline) and/or others considered as having *in vitro* activity as presented in Table 1. .

**Table 4:** Recommendations for the treatment of carbapenem-resistant *Acinetobacter baumannii* (CRAB).

**Infection site**	**Treatment options**	
LRT, SSTI, PBSI, IAA	**Preferential**	**Alternative**^d^^,^^e^
	Polymyxin B or colistin plus ampicillin-sulbactam^a^	Polymyxin B or colistin plus ampicillin-sulbactam^f^
	Polymyxin B or colistin plus tigecycline^a^^,^^b^	Polymyxin B or colistin plus tigecycline^g^
	Ampicillin-sulbactam plus tigecycline^a^^,^^b^	Ampicillin-sulbactam plus tigecycline^h^
	Polymyxin B or colistin OR ampicillin-sulbactam OR tigecycline plus ciprofloxacin OR levofloxacin OR TMP-SMX^a^^,^^b^	Polymyxin B or colistin plus meropenem^i^
	Ampicillin-sulbactam^c^	Polymyxin B or colistin plus aminoglycoside^j^
APN, cLUTI		
	Ciprofloxacin OR levofloxacin OR TMP-SMX OR ampicillin-sulbactamk, l	Polymyxin B or colistin plus ampicillin-sulbactam^f^^,^^m^
	Ciprofloxacin plus TMP-SMX^l^^l^	Polymyxin B or colistin plus meropenem^i^^,^^m^
	Ciprofloxacin plus ampicillin-sulbactam^l^^,^^l^	Polymyxin B or colistin plus aminoglycoside^m^^,^^j^
	Ampicillin-sulbactam plus TMP-SMX l^,^^l^	Tigecycline in combination^n^
uLUTI		
	Ciprofloxacin OR levofloxacin OR TMP-SMX OR ampicillin-sulbactam^o^	Polymyxin B or colistin^m^^,^^p^
		Amikacin, gentamicin, tobramycin^p^

APN, acute pyelonephritis; cLUTI, Ccomplicated lower urinary tract infection; LRTI, lower respiratory tract infection; IAI, intra-abdominal infection; PBSI, primary bloodstream infection; SSTI, skin and soft tissue infection; TMP-SMX, trimethoprim-Sulfamethoxazole; uLUTI; uncomplicated LUTI.

a Considering that all antimicrobials show *in vitro* activity as defined in Table 1, there is no clinical evidence of superiority of one combination regimen over another, nor of regimens containing polymyxins over regimens not containing polymyxins.

b Tigecycline exhibits low serum concentrations and is not recommended as a preferential treatment for primary bloodstream infections.

c Ampicillin-sulbactam can be used in monotherapy in selected patients with non-severe infections, provided *in vitro* activity with the appropriate methodology is confirmed, as recommended in Table 1, and doses are prescribed according to those recommended in Table 2.

d When at least one of the antimicrobials did not show *in vitro* activity as defined in Table 1.

e If no antimicrobials fall into these categories, combination therapy is still recommended; however, there is no evidence to recommend one antimicrobial over another.

f Preferred regimen provided that polymyxins present *in vitro* activity and ampicillin-sulbactam minimal inhibitory concentration (MIC) is within the range in which of potential *in vivo* activity through optimized dosing regimens.

g Preferred regimen provided that polymyxins present *in vitro* activity, ampicillin-sulbactam MIC is higher than that of potential *in vivo* activity through optimized dosing regimens or test is not available, and tigecycline MIC is within the range in which of potential *in vivo* activity through optimized dosing regimens.

h Preferred regimen when both drugs demonstrate potential *in vivo* activity and there is resistance to all other drugs. Meropenem can be added since synergism with sulbactam has been demonstrated.

i We suggest that a polymyxin be used in combination with meropenem provided it shows potential *in vivo* activity, and previous options are not possible. Ampicillin-sulbactam can be added since synergism between meropenem and sulbactam has been demonstrated.

j Aminoglycosides should be prescribed last due to their greater potential for nephrotoxicity in association with polymyxins.

k If categorized as susceptible, these antimicrobials should be considered as first-line agents, in non-critically ill patients.

l Combination is recommended for severe infections.

m For APN, cLUTI, and uLUTI, colistin is the preferential option, because of higher urinary concentrations compared to polymyxin B.

n Suggested only when previous alternatives are not possible, and owing to its lower urinary concentrations, tigecycline should always be combined with one or more agents to which potential *in vivo* activity may be achieved, as in Table 1.

o If categorized as susceptible, these antimicrobials should be considered as first-line agents.

p Accepted as monotherapy. The panel recommends combining only if there are no antimicrobial agents with *in vitro* activity, or in relapsed infections. Any other condition should be treated as cUTIs.

There is neither evidence indicating superiority of any specific combination over another, nor of polymyxins-containing schemes over schemes not including these drugs. Some preference for polymyxin-containing combinations is solely based on the susceptibility profile of CRAB isolates, which usually present MIC ≤2 mg/L for polymyxin B or colistin. However, the combination of ampicillin-sulbactam plus tigecycline is an option if MICs are ≤ 8/4 and ≤ 0.5 mg/L, respectively, and is the preferred regimen in the presence of resistance to polymyxins. Given the uncertainty regarding superiority of any regimen for CRAB, therapies should be chosen on an individual basis.

Ampicillin-sulbactam is one of the preferred drugs to be used in combination with a polymyxin due to its relatively low toxicity profile and availability in most hospitals. High dose tigecycline may be a preferred drug for combination with a polymyxin, if the MIC of tigecycline is ≤ 0.5 mg/L. However, either ampicillin-sulbactam or tigecycline MIC of ≤ 8/4 mg/L and ≤ 0.5 mg/L, respectively, are uncommon in CRAB isolates.

If no additional drug to combine with a polymyxin shows *in vitro* activity based on criteria presented in these guidelines (Table 1), the panel recommends that a drug with potential *in vivo* activity based on PK/PD profile should be added, with preference for those with the lowest MIC, *i.e.*, ampicillin-sulbactam of 16/8 mg/L or tigecycline of 1 mg/L.

The panel suggests meropenem as an option for combination, if ampicillin-sulbactam and tigecycline presented MICs > 64/32 mg/L and > 2 mg/L, respectively, and the MIC of meropenem is 16 or 32 mg/L. PK/PD studies show that high-dose extended infusion meropenem can achieve free concentration over these values 40 % of dose interval, considered the PK/PD target of meropenem, in a considerable proportion of patients.^45^, ^46^, ^47^, ^48^ In addition, free drug concentrations of meropenem >20 % and <40 % of the time can be achieved in a even higher proportion of patients, when the MIC of meropenem is 16 or 32 mg/L, and this PK/PD target was associated to static to 1 log colony-forming unit bacterial killing in an experimental study with neutropenic mouse.^49^ Although colistin in combination with meropenem were not beneficial over monotherapy in two RCTs, no evaluation according to the meropenem MIC was performed in these studies.^50^^,^^51^ Moreover, in one trial, meropenem was not used in optimized doses.^51^ Therefore, it should be regarded as a potential drug for combination in the absence of other options if meropenem is within this MIC range.

Combination of a polymyxin plus an aminoglycoside, if susceptible within brackets, is the last option based on the higher potential for acute kidney injury of this combination. Additionally, it is fundamental to state that there is neither PK/PD nor clinical evidence supporting the current breakpoints of aminoglycosides drugs for *A. baumannii*,^52^ in a way that the use of these drugs against CRAB is totally experimental. Nonetheless, it may be considered if none of the other antimicrobials present with MICs within the range of potential *in vivo* activity. The combination of three of the antimicrobial agents (preferebly including the ampicillin-sulbactam plus meropenem) may be considered in the presence of resistance to polymyxins.^78^

#### What are the recommendations for treatment of APN or cLUTI by CRAB?

These infections can be treated in monotherapy with a ciprofloxacin, TMP-SMX if there is *in vitro* susceptibility, and with ampicillin-sulbactam if the MIC is ≤8/4 mg/L. The combination of two antimicrobials is recommended if these conditions are not present. The panel also recommends the use of combination in severely ill or immunocompromised patients. For combination selection, consider the points presented for infections at non-urinary sites. If a polymyxin is chosen, colistin should be preferred considering its higher concentration in urine compared to that of polymyxin B. Owing to its lower concentration in urine, tigecycline should be regarded as the last option.

#### What are the recommendations for treatment of uLUTI by CRAB?

These infections can be treated in monotherapy with a single drug according to the results of ASTs. Polymyxins and aminoglycosides are acceptable in monotherapy if the isolate presents susceptibility within brackets, but ciprofloxacin, TMP-SMX and ampicillin-sulbactam are preferred options if *in vitro* susceptibility is observed, considering the higher potential for acute kidney injury of polymyxins and aminoglycosides.

The other considerations presented for the treatment of APN and cLUTI should be taken into account, when deciding the treatment of patients with uLUTI.

### Carbapenem-resistant *Pseudomonas aeruginosa*

#### What are the recommendations for treatment of RTI, SSTI, primary BSI and IAI by non-carbapenemase-producing CRPA?

Considering that non-carbapenemase-producing CRPA may present a broad variety of antimicrobial susceptibility profile, including *in vitro* activity to other beta-lactam agents, the choice of the best antimicrobial therapy may depend on this co-resistance profile (Table 5).

**Table 5:** Recommendations for the treatment of class A, class B, and non-carbapenemase producing (NC) carbapenem-resistant *Pseudomonas aeruginosa* (CRPA).

**Infection site**	**Treatment options (Carbapenemase type: A/B/NC or ANY type)**	
LRT, SSTI, PBSI, IAA	**Preferential**	**Alternative**
	Ceftolozane-tazobactam (NC)^a^	Polymyxin B or colistin combined with a second *in vitro* active agent^h^
	Ceftazidime-avibactam (A/NC)or imipenem-cilastatin-relebactam (A/NC)^b^^,^^c^	Ciprofloxacin, levofloxacin (ANY)^i^^,^^j^
	Ceftazidime-avibactam plus aztreonam (B)^d^^,^^e^^,^^f^	Ceftazidime, cefepime (NC)^i^
	Ceftazidime-avibactam plus aztreonam plus polymyxin B or colistin (B)^d^^,^^e^^,^^f^^,^^g^	Piperacillin-tazobactam (NC)^i^^,^^k^
		Imipenem-cilastatin-relebactam plus aztreonam (B)^l^
APN, cLUTI		
	Ceftolozane-tazobactam (NC)^a^	Ciprofloxacin, levofloxacin (ANY)^i^^,^^j^
	Ceftazidime-avibactam (A/NC)^b^^,^^c^ or imipenem-cilastatin-relebactam (A/NC)^b^^,^^c^	Ceftazidime, cefepime (NC)^i^
		Piperacillin-tazobactam (NC)^i^^,^^k^
	Ceftazidime-avibactam plus aztreonam (B)^d^^,^^e^^,^^f^	Amikacin, tobramycin (ANY)^n^
	Ceftazidime-avibactam plus aztreonam plus polymyxin B or colistin (B)^d^^,^^e^^,^^f^^,^^g^^,^^m^	Colistin or polymyxin B (ANY)^h^
		Imipenem-cilastatin-relebactam plus aztreonam (B)^l^
uLUTI		
	Ciprofloxacin, levofloxacin (ANY)^o^	Colistin or polymyxin B (ANY)^h^^,^^o^
	Amikacin, tobramycin (ANY)^o^	Imipenem-cilastatin-relebactam plus aztreonam (B)^l^
	Piperacillin-tazobactam (NC)^o^	
	Ceftolozane-tazobactam (NC)^a^	
	Ceftazidime-avibactam (A/NC)^b^^,^^c^ or imipenem-cilastatin-relebactam (A/NC)^b^^,^^c^	
	Ceftazidime-avibactam plus aztreonam (B)^o^	

APN, acute pyelonephritis; cLUTI, complicated urinary tract infection; LRTI, lower respiratory tract infection; IAI, intra-abdominal infection; PBSI, primary bloodstream infection; SSTI, skin and soft tissue infection; TMP-SMX, trimethoprim-sulfamethoxazole; uLUTI; uncomplicated LUTI.

a For severe infections and/or in neutropenic patients, ceftolozane-tazobactam should be considered the preferred treatment option for non-carbapenemase-producing isolates.

b The novel β-lactam/β-lactamase inhibitor combinations are considered first-line therapy for various *Pseudomonas aeruginosa* infections. However, their use should be rational, considering factors such as severity, inoculum, age, comorbidities (*e.g.*, renal dysfunction), and source control. In severe cases with hemodynamic instability and poor source control, these agents should be prioritized. For non-carbapenemase-producing isolates, ceftolozane-tazobactam is preferred to preserve ceftazidime-avibactam and imipenem-relebactam for treating carbapenemase-producing or difficult-to-treat resistant strains.

c There is no evidence supporting the preferential use of ceftazidime-avibactam over imipenem-relebactam, nor the combination of these agents with antimicrobials from different classes. Both should be used as monotherapy.

d Ceftazidime-avibactam has no activity against class B-producing isolates. Aztreonam is not hydrolyzed by class B, but can be hydrolyzed by other beta-lactamases produced by CRPA. The combination of ceftazidime-avibactam with aztreonam may restore the *in vitro* activity of aztreonam against class B carbapenemase-producing CRPA. Aztreonam should be infused simultaneously with ceftazidime-avibactam for best efficacy.

e The combination of ceftazidime-avibactam with aztreonam may be synergistic against certain CRPA isolates; however, its efficacy may vary depending on the specific β-lactamases produced and/or the presence of mutations in Penicillin-Binding Protein-3 (PBP3).

f The combination of ceftazidime-avibactam with aztreonam should be used against class B carbapenemase-producing CRPA without the addition of another antimicrobial agent, only if susceptibility testing indicates *in vitro* activity, using the breakpoint proposed in Table 1 (aztreonam-avibactam minimal inhibitory concentration [MIC], ≤ 4/4 g/L), or in cases of non-severe infections. Synergism of this combination can be tested by the disk elution technique, but this result does not substitute the results of aztreonam-avibactam susceptibility test.

g In severe infections caused by class B-producing CRPA and/or in patients with hemodynamic instability, the panel recommends that ceftazidime-avibactam combined with aztreonam be administered together with another antimicrobial agent (with a polymyxin B or colistin as the first option), if aztreonam-avibactam susceptibility testing is not available.

h We suggest that a polymyxin be used in combination with a second antimicrobial agent, preferably categorized as S, I, or (S), in that order of preference. If none are available, an antimicrobial with the possibility of rescuing the action *in vivo*, such as meropenem, if any of their respective Minimal Inhibitory Concentration (MIC) are within the range presented in Table 1. Aminoglycosides should be prescribed last due to their greater potential for nephrotoxicity. If no antimicrobials fall into these categories, combination therapy is still recommended; however, there is no evidence to recommend one antimicrobial over another. If MIC determination is not available, we recommend combining meropenem with other antibiotics.

i If a CRPA isolate is susceptible to narrower-spectrum agents (ceftazidime, cefepime, piperacillin-tazobactam, fluoroquinolones such as ciprofloxacin, levofloxacin), these should be preferred over other agents to avoid unnecessary exposure to broader-spectrum antibiotics.

j Fluoroquinolones, ciprofloxacin and levofloxacin, are the only oral antimicrobials available for treating less severe *P. aeruginosa* infections or for sequential therapy, and should preferably be prescribed to stable patients eligible for outpatient care. This phenotype is uncommon in CRPA, and evidence for fluoroquinolone monotherapy in severe systemic infections is limited.

k Broth microdilution is the recommended method for piperacillin-tazobactam MIC determination, as automated systems and gradient diffusion present unacceptably high rates of very major and minor errors.

l Although imipenem-cilastatin-relebactam also restores the activity of aztreonam against class B carbapenemase-producing isolates, it is not the preferred option because it has been tested in fewer isolates than ceftazidime-avibactam, and clinical data on the efficacy and safety of imipenem-relebactam are lacking.

m For APN, cLUTI, and uLUTI, colistin is the preferential option, because of higher urinary concentrations compared to polymyxin B. Combination therapy is recommended for APN and in cLUTI in critically ill patients.

n Aminoglycosides (amikacin, tobramycin) may be an option. The use of gentamicin is not recommended for the treatment of *P. aeruginosa*, and its clinical breakpoints have been removed from the BrCAST/EUCAST guidelines. Monotherapy with one of the recommended agents may be considered only for complicated but non-severe cLUTI in stable, non-critically ill patients. For APN or in critically ill patients, we recommend that aminoglycosides should be used in combination with a second antimicrobial agent preferably categorized as S, I or (S), in that order, or with the possibility of rescuing the *in vivo* action, such as meropenem, if they present with MICs described above. Polymyxins and aminoglycosides are the last preferred combination because of the greatest potential for nephrotoxicity.

o Accepted as monotherapy. The panel recommends combining only if there are no antimicrobial agents with *in vitro* activity, or in relapsed infections. Any other condition should be treated as cUTIs.

For non-carbapenemase-producing CRPA isolates with *in vitro* activity to beta-lactams (ceftazidime, cefepime, piperacillin-tazobactam, or aztreonam) or fluoroquinolones (ciprofloxacin or levofloxacin), the panel suggests that monotherapy with one of these narrower-spectrum agents for isolates should only be used in patients with less severe infections, who does neither present inadequate source control nor neutropenia. If susceptible, ceftazidime or cefepime are the preferred agents. Piperacillin-tazobactam may also be an option if the susceptibility test is confirmed by disk-diffusion or broth microdilution. Automated systems and gradient diffusion methods have demonstrated unacceptably high rates of very major errors (false susceptibility).^53^

For patients with severe infections, especially in critically ill patients, or in those with inadequate source control or neutropenia, the panel suggests that either ceftolozane-tazobactam or ceftazidime-avibactam are the preferred agents. The ceftolozane-tazobactam is preferred if the antimicrobial susceptibility test shows susceptibility to both drugs. Imipenem-relebactam may present *in vitro* activity against some isolates resistant to both ceftazidime-avibactam and ceftolozane-tazobactam due to mutations in AmpC or acquired OXA-10 variants.^54^^,^^55^ Therefore, imipenem-relebactam may be an option for these cases.

In the absence of one of these agents, a polymyxin combined with another agent with *in vitro* activity, either a beta-lactam or a fluoroquinolone (*e.g.*, ciprofloxacin or levofloxacin) should be used. Aminoglycosides (amikacin or tobramycin) may be used as combination therapy with a polymyxin if the potential for nephrotoxicity is acceptable, but this class is recommended only if there is no other agent with *in vitro* activity.

The panel recommends that caution should be taken with the use of a beta-lactam or a fluoroquinolone in monotherapy in isolates with resistance to meropenem, because this phenotype indicates the hyperexpression of efflux pumps that also affects these other agents. Hence, development of resistance during therapy may occur, either to increments in expression of efflux pumps or to derepression of AmpC, in the case of beta-lactams. Although these resistance mechanisms may also affect the activity of both ceftolozane-tazobactam and ceftazidime-avibactam, they are affected to a lesser degree. If there is only resistance to imipenem, but not to meropenem, this phenotype indicates that efflux pumps are not hyper-expressed,^56^ and the use of beta-lactams or fluoroquinolones might be considered as monotherapy for less severe infections in clinically stable patients.

If no antimicrobial other than a polymyxin presents *in vitro* activity, the panel recommends the use of a polymyxin combined with meropenem due to potential synergism, and the numerically higher survival rate observed in a double-blind randomized clinical trial.^51^

#### What are the recommendations for treatment of APN and cLUTI by non-carbapenemase-producing CRPA?

For patients with APN presenting with sepsis, the recommendations are the same as those for LRT, SSTI, PBSI and IAI.

For APN in stable patients who are not critically ill, as for cLUTIs, either a beta-lactam or a fluoroquinolone in monotherapy may be used as the first-choice therapy. Aminoglycosides in monotherapy are regarded as second line agents, but it is an alternative if the potential for nephrotoxicity is considered acceptable in stable patients with cLUTIs.

If no antimicrobial other than a polymyxin presents *in vitro* activity, the use of a polymyxin, preferably colistin, in monotherapy may be considered for cLUTIs in stable patients. In APN or in cLUTI with sepsis the panel recommends the combination with meropenem.

#### What are the recommendations for treatment of uLUTI by non-carbapenemase-producing CRPA?

For uLUTI monotherapy with an antimicrobial presenting *in vitro* activity can be used as the first option. A beta-lactam or a fluoroquinolone should be preferred, while aminoglycosides and polymyxins should be regarded as alternative agents, if other options did not present *in vitro* activity.

Although newer ceftolozane-tazobactam and ceftazidime-avibactam are recognized as first-line options for various CRPA infections, their use in uLUTI should be judicious and reserved for patients with renal dysfunction infected by isolates resistant to other beta-lactams and fluoroquinolones.

In cases of recurrence, the panel recommends that combination therapy with a polymyxin and another agent may be used, and the choice of the second agent should follow the same principles presented for systemic infections.

#### What are the recommendations for treatment of RTI, SSTI, PBSI and IAI by class A carbapenemase-producing by CRPA?

BL/BLIs combinations, either ceftazidime-avibactam or imipenem-relebactam, are considered the preferred options for treating systemic infections by class A carbapenemase-producing CRPA if *in vitro* susceptibility is demonstrated. Both agents are recommended as monotherapy, as no study has yet addressed the potential benefit of combining them with other antimicrobial classes. No direct clinical comparison is currently available between these two agents. Susceptibility testing for both BL/BLIs is essential to guide therapy, because there may be resistance to one and susceptibility to another.

If new BL-BLIs are not available, the panel recommends the use of a polymyxin combined with another agent with *in vitro* activity, either a fluoroquinolone or an aminoglycoside (amikacin or tobramycin). The potential for nephrotoxicity using aminoglycosides and polymyxins should be carefully evaluated.

If no antimicrobial other than a polymyxin presents *in vitro* activity, the panel recommends the use of a polymyxin combined with meropenem due to potential synergism, and the numerically higher survival rate observed in a double-blind randomized clinical trial.^51^

If fluoroquinolones (ciprofloxacin or levofloxacin) present *in vitro* activity, the panel suggests that monotherapy with any of these agents should only be used in patients with less severe infections, who does neither present inadequate source control nor neutropenia. However, this susceptibility phenotype is uncommon among carbapenemase-producing CRPA, and clinical evidence supporting fluoroquinolone monotherapy in this setting is limited.

#### What are the recommendations for treatment of APN or cLUTI by class A carbapenemase-producing CRPA?

For the treatment of APN and cLUTI caused by class A carbapenemase-producing CRPA, the recommendations align with those for other systemic infections. Monotherapy with either ceftazidime-avibactam or imipenem-relebactam is preferred. Monotherapy with ciprofloxacin may be selected in case-by-case selection as previously mentioned for systemic infections.

As alternative treatment options, monotherapy with once-daily aminoglycosides (amikacin or tobramycin) or polymyxins (colistin preferentially) may be considered for cLUTI in stable non-neutropenic patients.

#### What are the recommendations for treatment of RTI, SSTI, PBSI and IAI by class B carbapenemase-producing by CRPA?

The panel recommends that a polymyxin combined with ceftazidime-avibactam plus aztreonam is the preferred regimen for severe systemic infections, particularly in critically ill or neutropenic patients.

Although the combination of ceftazidime-avibactam plus aztreonam may demonstrate *in vitro* activity against class B carbapenemase-producing CRPA, owing to the current paucity of clinical studies evaluating these combinations for *P. aeruginosa*,^57^ the panel suggests the use of these drugs without the combination of a polymyxin should be conditioned to the following situations: i) In less severe infections and stable patients if MIC testing for aztreonam-avibactam of ≤4/4 mg/L, based on current breakpoint for *Enterobacterales*; or ii) In less severe infections and stable patients if MIC testing for aztreonam-avibactam of 8/4 mg/L, and exceptionally of 16/4 mg/L, based on current aztreonam alone breakpoints, in patients who have failed to the treatment with or is unable tolerate a polymyxin. There is no study correlating *in vitro* synergy tests neither with lower MICs of aztreonam-avibactam nor with clinical outcomes. Therefore, the panel does not recommend that a synergy testing may substitute the MIC determination for the evaluation of the potential *in vitro* activity. *In vitro* activity of aztreonam-avibactam may be compromised in the presence of penicillin-binding protein 3 (PBP3) mutations, or depending on the specific beta-lactamases produced by the CRPA isolate.^58^^,^^59^ Therefore, in the absence of MIC determination for aztreonam-avibactam, the use of ceftazidime-avibactam plus aztreonam without a polymyxin, or occasionally of another potential drug showing *in vitro* activity (such as a fluoroquinolone or an aminoglycoside) should only be considered for non-severe infections in patients whose the anticipated consequences of a therapeutic failure to the initial scheme are tolerable.

Notably, in some isolates aztreonam alone may present *in vitro* activity. In these cases, the panel suggests that aztreonam may be the preferential agent for combination therapy with a polymyxin. As monotherapy, aztreonam may only be considered for stable patients with no severe infections.

If ceftazidime-avibactam plus aztreonam are not available, the panel recommends the use of a polymyxin combined with another agent with *in vitro* activity, either a fluoroquinolone or an aminoglycoside (amikacin or tobramycin). The potential for nephrotoxicity using aminoglycosides and polymyxins should be carefully evaluated. If no antimicrobial other than a polymyxin presents *in vitro* activity, the panel recommends the use of a polymyxin combined with meropenem for the same reason discussed for other CRPA mechanisms.^51^

If fluoroquinolones (ciprofloxacin or levofloxacin) present *in vitro* activity, the panel suggests that monotherapy with any of these agents should only be used in patients with less severe infections, who does neither present inadequate source control nor neutropenia. However, as for CRPA with other carbapenem resistance mechanisms, this susceptibility phenotype is uncommon and clinical evidence supporting fluoroquinolone monotherapy in this setting is limited.

#### What are the recommendations for treatment ofAPN or cLUTI by class B carbapenemase-producing CRPA?

For patients with APN presenting with sepsis, the recommendations are the same as those for LRT, SSTI, PBSI and IAI.

For APN in stable patients who are not critically ill, as for cLUTIs, the panel suggests that either aztreonam or a fluoroquinolone in monotherapy may be used as the first-choice therapy. For these patients, ceftazidime-avibactam plus aztreonam without the addition of another agent may be an option even without MIC testing for aztreonam-avibactam, if the isolate is resistant to fluoroquinolones and aztreonam alone. However, the addition of a polymyxin (preferably, colistin) or an aminoglycoside may be a better alternative in more severe infections.

If no antimicrobial other than a polymyxin presents *in vitro* activity, and ceftazidime-avibactam plus aztreonam is not available, the use of a polymyxin, preferably colistin, in monotherapy may be considered for cLUTIs in stable patients. In APN or in cLUTI with sepsis the panel recommends the combination of a polymyxin with meropenem.

As alternative treatment options, monotherapy with once-daily aminoglycosides (amikacin or tobramycin) or polymyxins (colistin preferentially) may be considered for cLUTI in stable non-neutropenic patients.

#### What are the recommendations for treatment of uLUTIs by carbapenemase-producing CRPA?

For uLUTI monotherapy with an antimicrobial presenting *in vitro* activity can be used as the first option. The panel recommends that aztreonam (for class B carbapenemase-producing) or a fluoroquinolone (regardless of the mechanism) should be preferred, while aminoglycosides and polymyxins should be regarded as alternative agents, if other options did not present *in vitro* activity.

Although ceftazidime-avibactam alone or with aztreonam are recognized as first-line options for various CRPA infections caused by isolates producers of class A or Class B carbapenemases, respectively, their use in uLUTI should be judicious and reserved for patients with renal dysfunction infected by isolates resistant to aztreonam alone (in class B carbapenemase-producing isolates).

In cases of recurrence, the panel suggests that combination therapy with a polymyxin and another agent may be used, and the choice of the second agent should follow the same principles presented for systemic infections.

### ESBL-producing enterobacterales (ESBL-E) and AmpC-producing *Enterobacterales* (AmpC-E)

#### What are the recommendations for treatment of RTI, SSTI, PBSI and IAI by ESBL-E and AMPC-E?

Carbapenems are the drugs of choice for the treatment of systemic infections, including LRT, SSTI, PBSI, and IAA, caused by ESBL-E and AmpC-E (Table 6). In critically ill patients or those with hypoalbuminemia, meropenem or imipenem are preferred over ertapenem. Cefepime should be preferred over carbapenems for systemic infections caused by AmpC-E isolates that are susceptible to third-generation cephalosporins. The panel suggests that cefepime may also be occasionally an option in less severe infections caused by AmpC-E isolates resistant to third-generation cephalosporins (Table 6); however, close monitoring of possible selection of fully derepressed AmpC is warranted. The panel emphasizes that cefepime is not an alternative for the treatment of infections caused by ESBL-E.

**Table 6:** Recommendations for the treatment of class A extended-spectrum beta-lactamase-producing *Enterobacterales* (ESBL-E) and *Enterobacterales* carrying chromosomal AmpC (AmpC-E).a.

**Infection site**	**Treatment options**	
LRT, SSTI, PBSI, IAA	**Preferential**	**Alternative**
	Meropenem, imipenem or ertapenem (ESBL-E and AmpC-E)^b^	Ciprofloxacin or levofloxacin (ESBL-E and AmpC-E)^d^
	Cefepime (AmpC-E)^c^	Ceftolozane-tazobactam (ESBL-E)^e^
		Ceftazidima-avibactam, imipenem-cilastatin-relebactam^f^
APN, cLUTI		
	Ciprofloxacin or levofloxacin (ESBL-E and AmpC-E)^b^^,^^g^ Meropenem, imipenem or ertapenem (ESBL-E and AmpC-E) Cefepime (AmpC-E)^i^ TMP-SMX (ESBL-E and AmpC-E)^j^	Amikacin, gentamicin, tobramycin (ESBL-E and AmpC-E)^k^
		Ceftolozane-tazobactam (ESBL-E)^e^
		Ceftazidime (ESBL-E and AmpC-E)^l^
		Ceftriaxona (AmpC-E)^m^
		Piperacillin-tazobactam (ESBL-E)^n^
uLUTI		
	Fosfomycin, nitrofurantoin (ESBL-E and AmpC-E)^o^	Amikacin, gentamicin, tobramycin (ESBL-E and AmpC-E)^k^
	Ciprofloxacin, levofloxacin, TMP-SMX (ESBL-E and AmpC-E)^p^	Ceftazidime (ESBL-E and AmpC-E)^l^
		Amoxicillin-clavulanate, piperacillin-tazobactam (ESBL-E)^n^^,^^q^

APN, acute pyelonephritis; cLUTI, complicated urinary tract infection; LRTI, lower respiratory tract infection; IAI, intra-abdominal infection; PBSI, primary bloodstream infection; SSTI, skin and soft tissue infection; TMP-SMX, trimethoprim-sulfamethoxazolem; uLUTI; uncomplicated LUTI.

a ESBL-E: *Klebsiella pneumoniae, E. coli*, and *Proteus mirabilis*. AmpC-E: *Enterobacter cloacae, Klebsiella aerogenes, Citrobacter freundii, Serratia marcescens, Morganella morganii,* and *Providencia stuartii.*

b Meropenem or imipenem are preferred in critically ill patients and those with hypoalbuminemia, for both ESBL-E and AmpC-E.

c Cefepime should be preferred over carbapenems for 3rd generation cephalosporin-susceptible AmpC-E, and for less severe infections caused by 3rd generation cephalosporins-resistant AmpC-E.

d Fluoroquinolones may be considered in mild infections, or as an option for oral switch in clinically stable patients.

e Although ceftolozane-tazobactam may be an effective option for the treatment of ESBL-E infections, we recommend that it should be reserved for the treatment of *P. aeruginosa* or mixed (ESBL + *P. aeruginosa*) infections. Ceftolozane-tazobactam has limited activity against AmpC-E.

f Although ceftazidime-avibactam and imipenem-relebactam have activity against ESBL-E and AmpC-E isolates, these antimicrobials should be reserved for the treatment of carbapenem-resistant Gram-negative infections.

g Fluoroquinolones are the preferred drugs for non-critically ill patients with either APN or cLUTI.

i Cefepime should be preferred over carbapenems for 3rd generation cephalosporin-susceptible AmpC-E even in critically ill patients, as for the treatment of 3rd generation cephalosporin-resistant AmpC-E APN/cLUTI in non-critically ill patients.

j TMP-SMX is the preferred drug for non-critically ill patients with cLUTI, and may be an alternative in non-critically ill patients with APN.

k When the potential for nephrotoxicity is considered acceptable, aminoglycosides are second-line options, in stable, non-critically ill patients.

l Ceftazidime may be susceptible in CTX-M-2-producing ESBL-E; however, no clinical data is available. We recommend it as a carbapenem-sparing alternative in stable non-critically ill patients with urinary tract infections if other options are not possible. Ceftazidime may also be an alternative in stable, non-critically ill patients with urinary tract infections by 3rd generation cephalosporin-susceptible AmpC-E. It is neither recommended for ESBL-E nor 3rd generation cephalosporin-susceptible AmpC-E for APN complicated with abscess, or in any other condition in which a higher inoculum may be present.

m Ceftriaxone may be an alternative in stable, non-critically ill patients with urinary tract infections by 3^rd^ generation cephalosporin-susceptible AmpC-E, with the same caveats presented for ceftazidime. Ceftazidime is preferred over ceftriaxone because of the non-urinary excretion of ceftriaxone.

n Piperacillin-tazobactam may be an alternative in stable, non-critically ill patients against urinary tract infections by ESBL-E. This combination is not recommended for AmpC-E.

o Both are first options only for *E. coli*.

p Fluoroquinolone or TMP-SMX should be considered as first-line agents against non-*E. coli* isolates. If the isolate is not susceptible to these agents, consider the options presented for APN/cLUTI.

q Amoxicillin-clavulanate may be an alternative for ambulatory treatment of uLUTI by ESBL-E.

Fluoroquinolones may be considered alternative agents for the treatment of milder infections in stable patients caused by ESBL-E and AmpC-E, or used as a step-down option for oral therapy in patients showing clinical response to initial treatment. Although, ceftolozane-tazobactam may be effective against ESBL-E infections,^60^ the panel recommends reserving this drug for the treatment of *P. aeruginosa* or co-infections with ESBL-E. A clinical trial was designed to better define the role of ceftolozane-tazobactam compared to meropenem for the treatment of BSI caused by third-generation cephalosporin-non-susceptible *Enterobacterales* or known chromosomal AmpC-producing *Enterobacterales*. However, this trial was withdrawn. In this way, it is important to note that ceftolozane-tazobactam has not been recommended against AmpC-E.

Although ceftazidime-avibactam and imipenem-relebactam are active against ESBL-E and AmpC-E isolates, these drugs should not be used to treat such bacteria and should instead be reserved for the treatment of carbapenem-resistant Gram-negative organisms.

#### What are the recommendations for treatment of APN and cUTI by ESBL-E and AMPC-E?

In the treatment of APN or cLUTI, fluoroquinolones, either ciprofloxacin or levofloxacin, can be used whenever susceptibility is confirmed against ESBL-E and AmpC-E isolates. However, especially in critically ill or hemodynamically unstable patients, meropenem may be preferred. Likewise, cefepime can be used for AmpC-E infections in critically ill patients when isolates are susceptible to third generation cephalosporins. TMP-SMX may be used in cLUTI cases with confirmed susceptibility, and occasionally in non-critical patients with acute pyelonephritis (Table 6).

Aminoglycosides are reserved as alternatives to first-line drugs, even in the treatment of UTIs, whenever the potential for nephrotoxicity is considered acceptable, since patients with UTIs treated with these drugs in monotherapy tended to present higher clinical failure and had significantly higher microbiological failures compared to other drugs, in a meta-analysis of randomized clinical trials.^61^

It is important to note that ceftazidime may appear susceptible in ESBL-producing isolates, particularly those producing CTX-M enzymes. However, there are currently no clinical data supporting its use in this setting. The panel suggests that ceftazidime may be considered as a carbapenem-sparing option in non-critically ill patients with cUTIs, if other options are unavailable or not feasible for the patient. Ceftazidime may also be used for non-severe cUTI caused by AmpC-E isolates, provided they are susceptible to both third-generation cephalosporins. Ceftazidime is preferred over ceftriaxone due to its urinary excretion profile. However, the panel does not recommend third-generation cephalosporins for patients with UTIs whenever abscesses are present. The panel emphasizes that third-generation cephalosporins are not recommended for AmpC-E isolates causing systemic infections.

The panel considers that piperacillin-tazobactam may be an alternative for non-critically ill, stable patients with urinary tract infections caused by ESBL-E. However, it is not recommended for patients with more severe infections. This combination is also not recommended for the treatment of AmpC-E infections, and alternative therapies should always be sought.^62^ However, if it is considered in a specific case as a therapeutic option for the treatment of severe ESBL- or AmpC-producing infections, the panel recommends confirming piperacillin-tazobactam MICs by using broth microdilution when this agent is intended to be prescribed for the treatment of severe ESBL- or AmpC-producing infections. False susceptibility to piperacillin-tazobactam has been observed with automated systems, gradient strips, and disk diffusion methods compared to broth microdilution, particularly in ESBL producers that co-produce OXA-1 (a narrow-spectrum oxacillinase) and in AmpC producers. Additionally, a significant increase in piperacillin-tazobactam MICs has been reported when testing high inocula against both ESBL and non-ESBL producers.^63^^,^^64^

#### What are the recommendations for treatment of uLUTI by Esbl-E and AMPC-E?

For uncomplicated urinary tract infections, fosfomycin and nitrofurantoin can be used for *E. coli*, and preference should be given to oral agents such as ciprofloxacin, levofloxacin, or TMP-SMX.

Alternative agents are the same as those for cLUTIs. The panel considers that oral beta-lactamase inhibitor combinations, such as amoxicillin-clavulanate, may be used as an option for ambulatorial patients (Table 6).

### *Stenotrophomonas maltophilia*

AST for *S. maltophilia* remains challenging. According to the BrCAST document, clinical breakpoints have been established only for TMP-SMX.^19^ Moreover, there is no universally accepted standard-of-care antibiotic regimen for the treatment of *S. maltophilia* infections.^5^ This lack of standardization, combined with the absence of clinical trials evaluating commonly used agents, limits the ability to compare therapeutic efficacy. Consequently, current data are insufficient to establish a clear preference among drugs with *in vitro* activity or to determine the added value of combination therapy. However, observational data indicate that patients treated with one of the available options have lower risk for mortality than patients who did not receive appropriate antimicrobial therapy.^65^

In Brazil, the antimicrobials commercially available for clinical use with *in vitro* activity against *S. maltophilia* include ceftazidime-avibactam, aztreonam, levofloxacin, tigecycline, and TMP-SMX. For the treatment of moderate and severe infections or treatment of infections in immunocompromised hosts, combination therapy has been a recommended strategy, although clinical evidence supporting this recommendation is limited (Table 7). The preferred therapeutic options include TMP-SMX combined with either tigecycline or levofloxacin (Table 7). While no current evidence supports the superiority of one agent over the other, it is important to consider PK/PD properties. Specifically, the panel suggests that tigecycline should be used with caution and only as part of combination therapy in the treatment of PBSIs.

**Table 7:** Recommendations for the treatment of *Stenotrophomonas maltophilia* and *Burkholderia cepacia* complex.

Treatment options	
*Stenotrophomonas maltophilia*	
Preferential	Alternative
TMP-SMX ± levofloxacin^a^^,^	Ceftazidime-avibactam plus aztreonam^b^^,^^c^
TMP-SMX ± tigecycline^a^	Ceftazidime-avibactam plus aztreonam ± TMP-SMX or levofloxacin or tigecycline^c^
Levofloxacin ± tigecycline^a^	Polymyxin B or colistin^d^
*Burkholderia cepacia* complex	
Preferential	Alternative
TMP-SMX ± ceftazidime or meropenem^e^	Ceftazidime-avibactam ± TMP-SMX or levofloxacin^f^
TMP-SMX ± levofloxacin ± tigecycline^e^	
Levofloxacin ± ceftazidime or meropenem^e^	

TMP-SMX, trimethoprim-sulfamethoxazole.

a The use of combination therapy is recommended for moderate to severe infections. Consider monotherapy with one *in vitro* active agent for mild infections.

b Ceftazidime-avibactam plus aztreonam may be an alternative in patients with more severe disease, contraindication to the use of other antimicrobials, or therapeutic failure to initial regimens, ensuring it has *in vitro* activity according to Table 1 definitions. If a susceptibility test is not available, the use of a combination with one of the preferential agents showing *in vitro* activity is recommended. Combination may also be considered in more severe infections.

c *In vitro* activity of ceftazidime-avibactam plus aztreonam cannot be inferred from synergism testing only. Combination with a second agent showing *vitro* activity according to Table 1 definitions may also be considered in more severe infections.

d *S. maltophilia* is not intrinsically resistant to polymyxins. However, minimal inhibitory concentrations above 2 mg/L are frequent. No clinical experience reported. Use a polymyxin for *S. maltophilia* infections, only if all other alternatives are not available.

e Combination of two agents is suggested if minimal inhibitory concentrations (MICs) are close or at the breakpoint suggested for *in vitro* activity, considering the lower accuracy of MIC in Bcc isolates. Combination should also be considered in severe infections and when MIC determination is not possible.

f The addition of avibactam does not reliably restore ceftazidime activity against *Bcc* because resistance involves multiple mechanisms (efflux pumps, low permeability, altered PBPs, and β-lactamase variants like PenA, many of which are not affected by avibactam. Consider combination therapy in the same situations described above.

If none of the above recommended options shows *in vitro* activity (Table 1), the combination of ceftazidime-avibactam plus aztreonam may be considered in patients with severe *S. maltophilia* infections. In addition, this is an alternative for patients who are unable to tolerate first-line agents or have experienced treatment failure with TMP-SMX – based regimens. Ideally, while no other antimicrobial susceptibility test is available, MIC of aztreonam-avibactam should be determined to support this therapy in severe infections, if the results are within the values proposed in Table 1. As for other class B-carbapenemase producing GNB, it is important to have in mind that *in vitro* activity of this combination without testing is only presumed based on resistance mechanisms involved in carbapenem resistance. The use of ceftazidime-avibactam plus aztreonam without a third agent should be limited to non-severe infections or cases where susceptibility testing confirms activity to this combination, defined by an aztreonam-avibactam MIC of ≤4/4 or, occasionally of 8 or 16/4 mg/L. Importantly, susceptibility to aztreonam-avibactam cannot be inferred from synergy testing alone. If employed as part of a triple combination regimen, this therapy should ideally include one of the preferred treatment options.

### *Burkholderia cepacia* complex (Bcc)

BrCAST/EUCAST do not present clinical breakpoints for any antimicrobials in their documents. In addition, current international guidelines lack specific treatment recommendations for Bcc infections. Considering there is a very limited number of studies in patients without cystic fibrosis evaluating antimicrobial therapies and strategies,^66^, ^67^, ^68^, ^69^, ^70^ the panel recommends managing severe Bcc infections based on MIC results, given their potential for *in vitro* activity (Table 1). Low accuracy in MIC determination has been reported for this pathogen.^71^^,^^72^ However, most isolates presented agreement within an 1 log_2_ dilution in studies that evaluated MIC methods, including when gradient strip was compared to BMD.^71^^,^^72^ Clinicians should take this information into account when interpreting MIC of Bcc results.

Based on *in vitro* activity according to definitions of this document, the panel recommends the use of at least one of the following agents classified as having *in vitro* activity: TMP-SMX, meropenem, ceftazidime, or levofloxacin (Table 7). Combination of two agents may be preferred, especially if MICs are close or at the cutoff suggested for *in vitro* activity, considering the lower accuracy of MIC in Bcc isolates. Combination should also be considered in severe infections and when MIC determination is not possible.

Case reports have documented successful treatment with ceftazidime-avibactam; however, there is no current evidence supporting its use as monotherapy or in combination regimens. Noteworthy, despite the presence of known class A and class C beta-lactamases in Bcc, variable capacity of avibactam to enhance ceftazidime activity has been demonstrated, suggesting that efflux-mediated resistance mechanisms may overcome the benefit of beta-lactamase inhibition.^73^^,^^74^ Therefore, determining the MIC of ceftazidime-avibactam in Bcc is necessary to ensure a lower MIC with the addition of avibactam and infer a potential clinical benefit.

### Infections in difficult to treat sites

Infections caused by MDR-GNB in difficult-to-treat sites, such as central nervous system infections, osteomyelitis, and infectious endocarditis, are limited to case reports or small case series. No studies are available comparing different therapies for infections in these sites.

Overall, the panel recommends that, whenever such infections occur, first-line treatment options should be prioritized. Appropriate surgical management when indicated is mandatory, and the limitations of antimicrobial therapy for some MDR-GNB should be clearly presented to surgeons when evaluating these patients. Furthermore, it should be noted that treatment durations in these infections are usually longer.

The panel recommends that, in addition to the above considerations, maximum dosing should be applied. If first-line therapies are not feasible, management by an infectious diseases’ physician is strongly recommended.

The intrathecal or intraventricular administration of antibiotics with poor cerebrospinal fluid penetration, such as polymyxins,^75^ aminoglycosides,^76^ and even tigecycline,^77^ may be necessary for the management of patients with meningitis and ventriculitis.

## Final considerations

The guideline's recommendations are grounded in the best available evidence and reflect the current availability of antimicrobial agents in Brazil. Given the complexity of MDR infections and the variability in clinical scenarios, the panel recommends that treatment decisions be individualized and, when appropriate, guided by consultation with a specialist in clinical microbiology and antimicrobial therapy. Such complexities are often not detailed in current guidelines, and it is very unlikely that there will ever be clinical studies encompassing all possible nuances that MDR-GNB isolates, and patients infected by these microorganisms may present. Therefore, some recommendations were based on the panel's expert opinion, aiming to present some guidance for most challenging situations that are faced in daily clinical practice of physicians treating patients with MDR-GNB infections.

MDR-GNB infections remain highly prevalent in Brazilian healthcare settings, largely driven by the spread of high-risk bacterial clones. Enhanced implementation of infection prevention and control strategies, coupled with expanded access to rapid diagnostic methods and essential antimicrobials across both public and private healthcare systems, could substantially reduce the burden of MDR infections in our country.

Despite the approval of several novel agents by international regulatory bodies such as the EMA and FDA, many of these antimicrobials remain inaccessible in Brazil due to limited commercial interest and/or delays in national regulatory approval. Additionally, the panel points out that the few novel antimicrobial agents that are commercially available in Brazil comprise the first line therapeutic options for most carbapenem-resistant GNB, due to its clinical benefits demonstrated by lower mortality rates and decreased toxicity. However, these novel agents are not available for the treatment of patients infected by MDR-GNB in the majority of public hospitals, in which therapies mostly rely on less potent and more toxic antimicrobials. A key barrier to the adoption of new antimicrobials and diagnostic technologies is the absence of local cost-effectiveness studies that demonstrate their potential to reduce mortality, lenght of hospitalization, and the transmission of resistant pathogens. In conclusion, this document is intended not only as a practical guide to inform clinical decision-making, but also as an advocacy tool to promote broader access to effective diagnostics and antimicrobial therapies.

## Conflicts of interest

The following authors have received honoraria for lectures and/or educational activities: AC (MSD, Pfizer); ACG (BioMérieux, Eurofarma, MSD, Pfizer, Roche, Sandoz); CAC (Apsen, Astellas, AstraZeneca, Basilea Pharmaceutica, Bayer, Cerexa, Daiichi-Sankyo, Dr Reddy’s, Eli Lilly, Eurofarma, GlaxoSmithKline, Janssen-Cilag, MSD, Novartis, Novo Nordisk, Pfizer, Sanofi Aventis, Teva, United Medical); DWCLS (BD, BioMérieux, Mundipharma, United Therapeutics); and GCT (Eurofarma, GlaxoSmithKline, MSD, Pfizer). All other authors declare no conflicts of interest.
